# Supplementary material for: Confining H3PO4 network in covalent organic frameworks enables proton super flow
Source: Nat Commun. 2020 Apr 24;11:1981. doi: 10.1038/s41467-020-15918-1 (PMC7181855; doi:10.1038/s41467-020-15918-1)
Supplement: Supplementary file 1 — Supplementary Information [file 41467_2020_15918_MOESM1_ESM.pdf]

# Supplementary Information

## Confining $H_3PO_4$ network in covalent organic frameworks enables proton super flow

Shanshan Tao,[1] Lipeng Zhai,[1] A. D. Dinga Wonanke,[2] Matthew A. Addicoat,[2] Qiuhong Jiang[1] & Donglin Jiang[1,3]★

[1]*Department of Chemistry, Faculty of Science, National University of Singapore, 3 Science Drive 3, Singapore 117543, Singapore.*

[2]*School of Science and Technology, Nottingham Trent University, Clifton Lane, Nottingham, NG11 8NS, UK.*

[3]*Joint School of National University of Singapore and Tianjin University, International Campus of Tianjin University, Binhai New City, Fuzhou 350207, China.*

*Corresponding author. Email: chmjd@nus.edu.sg

**Contents**

**Supporting Tables** (Pages S2-S6)

**Supporting Figures** (Pages S7-S24)

**Supplementary Tables**

**Supplementary Table 1. Atomistic coordinates for the AA-stacking mode of TPB-DMeTP-COF (space group $P_6$, $a = b = 37.2718$ Å, $c = 3.52$ Å, $\alpha = \beta = 90°$ and $\gamma = 120°$).**

| Atom | *x/a* | *y/b* | *z/c* |
|---|---|---|---|
| C1 | 0.28976 | 0.64139 | 0.55868 |
| C2 | 0.31518 | 0.62343 | 0.55861 |
| C3 | 0.24365 | 0.61467 | 0.56057 |
| C4 | 0.37304 | 0.5916 | 0.39498 |
| C5 | 0.39771 | 0.57323 | 0.39744 |
| C6 | 0.43546 | 0.5919 | 0.56888 |
| C7 | 0.44834 | 0.62976 | 0.72965 |
| C8 | 0.4234 | 0.6478 | 0.7269 |
| N9 | 0.46243 | 0.57524 | 0.57192 |
| C10 | 0.44612 | 0.53497 | 0.57207 |
| C11 | 0.47306 | 0.51647 | 0.56812 |
| C12 | 0.45639 | 0.47284 | 0.56762 |
| C13 | 0.48356 | 0.45722 | 0.56726 |
| H14 | 0.30091 | 0.58941 | 0.55889 |
| H15 | 0.34391 | 0.57707 | 0.25659 |
| H16 | 0.38812 | 0.54461 | 0.25932 |
| H17 | 0.4777 | 0.64432 | 0.86445 |
| H18 | 0.43332 | 0.67687 | 0.85963 |
| H19 | 0.41162 | 0.51332 | 0.58125 |
| H20 | 0.4713 | 0.42344 | 0.5676 |
| C21 | 0.58995 | 0.55686 | 0.56749 |
| H22 | 0.60476 | 0.55232 | 0.35371 |
| H23 | 0.60478 | 0.55238 | 0.78144 |
| H24 | 0.59664 | 0.58914 | 0.56721 |

**Supplementary Table 2. Atomistic coordinates for TPB-DMeTP-COF via Pawley refinement (space group $P_6$, $a = b = 36.5281$ Å, $c = 3.41249$ Å, $\alpha = \beta = 90°$ and $\gamma = 120°$).**

| Atom | *x/a* | *y/b* | *z/c* |
|---|---|---|---|
| C1 | 0.29278 | 0.64806 | 0.55868 |
| C2 | 0.31846 | 0.62991 | 0.55861 |
| C3 | 0.24618 | 0.62106 | 0.56057 |
| C4 | 0.37692 | 0.59776 | 0.39498 |
| C5 | 0.40185 | 0.57919 | 0.39744 |
| C6 | 0.43999 | 0.59806 | 0.56888 |
| C7 | 0.453 | 0.63631 | 0.72965 |
| C8 | 0.4278 | 0.65454 | 0.7269 |
| N9 | 0.46724 | 0.58122 | 0.57192 |
| C10 | 0.45077 | 0.54054 | 0.57207 |
| C11 | 0.47798 | 0.52185 | 0.56812 |
| C12 | 0.46114 | 0.47776 | 0.56762 |
| C13 | 0.48859 | 0.46198 | 0.56726 |
| H14 | 0.30404 | 0.59554 | 0.55889 |
| H15 | 0.34749 | 0.58307 | 0.25659 |
| H16 | 0.39216 | 0.55027 | 0.25932 |
| H17 | 0.48267 | 0.65102 | 0.86445 |
| H18 | 0.43783 | 0.68392 | 0.85963 |
| H19 | 0.4159 | 0.51866 | 0.58125 |
| H20 | 0.4762 | 0.42785 | 0.5676 |
| C21 | 0.59609 | 0.56265 | 0.56749 |
| H22 | 0.61105 | 0.55806 | 0.35371 |
| H23 | 0.61107 | 0.55812 | 0.78144 |
| H24 | 0.60285 | 0.59527 | 0.56721 |

**Supplementary Table 3. Atomistic coordinates for TPB-DMeTP-COF via Rietveld refinement (space group $P_6$, $a = b = 37.6497$ Å, $c = 3.4662$ Å, $\alpha = \beta = 90°$ and $\gamma = 120°$).**

| Atom | *x/a* | *y/b* | *z/c* |
|---|---|---|---|
| C1 | 0.29302 | 0.6486 | 0.55868 |
| C2 | 0.31873 | 0.63044 | 0.55861 |
| C3 | 0.24639 | 0.62158 | 0.56057 |
| C4 | 0.37723 | 0.59826 | 0.39498 |
| C5 | 0.40218 | 0.57967 | 0.39744 |
| C6 | 0.44036 | 0.59856 | 0.56888 |
| C7 | 0.45338 | 0.63684 | 0.72965 |
| C8 | 0.42816 | 0.65509 | 0.7269 |
| N9 | 0.46763 | 0.58171 | 0.57192 |
| C10 | 0.45114 | 0.54099 | 0.57207 |
| C11 | 0.47838 | 0.52228 | 0.56812 |
| C12 | 0.46152 | 0.47816 | 0.56762 |
| C13 | 0.489 | 0.46236 | 0.56726 |
| H14 | 0.30429 | 0.59604 | 0.55889 |
| H15 | 0.34778 | 0.58356 | 0.25659 |
| H16 | 0.39249 | 0.55073 | 0.25932 |
| H17 | 0.48307 | 0.65157 | 0.86445 |
| H18 | 0.4382 | 0.68449 | 0.85963 |
| H19 | 0.41625 | 0.51909 | 0.58125 |
| H20 | 0.4766 | 0.42821 | 0.5676 |
| C21 | 0.59659 | 0.56312 | 0.56749 |
| H22 | 0.61156 | 0.55853 | 0.35371 |
| H23 | 0.61158 | 0.55859 | 0.78144 |
| H24 | 0.60335 | 0.59577 | 0.56721 |

**Supplementary Table 4. BET surface area, Langmuir surface area, pore size and pore volume of TPB-DMeTP-COF after treatment under different conditions.**

| TPB-DMeTP-COF | BET surface area ($m^2\ g^{-1}$) | Langmuir surface area ($m^2\ g^{-1}$) | Pore size (nm) | Pore volume ($cm^3\ g^{-1}$) |
|---|---|---|---|---|
| **As-synthesized** | 2894 | 4586 | 3.36 | 1.60 |
| **THF 7 days** | 2900 | 4596 | 3.36 | 1.51 |
| **$H_3PO_4$ 7 days** | 2703 | 4283 | 3.36 | 1.42 |
| **$CH_3CN$ 7 days** | 2895 | 4588 | 3.36 | 1.41 |
| **Water, 25 °C 7 days** | 2736 | 4336 | 3.36 | 1.38 |
| **Water, 100 °C 7 days** | 2726 | 4320 | 3.36 | 1.37 |
| **HCl, 12 M 7 days** | 2693 | 4268 | 3.36 | 1.32 |
| **NaOH, 14 M 7 days** | 2710 | 4295 | 3.36 | 1.33 |
| **Fenton's reagent 1 day** | 2830 | 4484 | 3.36 | 1.41 |
| **After AC impedance** | 2292 | 3632 | 3.33 | 1.22 |

**Supplementary Table 5. Molecular dynamics calculations of $^{1}H$ and $^{31}P$ diffusion coefficients of $H_3PO_4$ in $H_3PO_4$@TPB-DMeTP-COF using DFTB (mio-0-1, NVE, 383 K).**

| | $D_H$ ($cm^2$ $s^{-1}$) | $D_P$ ($cm^2$ $s^{-1}$) |
|---|---|---|
| **$H_3PO_4$@TPB-DMeTP-COF** | $6.28 \times 10^{-4}$ | $3.26 \times 10^{-4}$ |

## Supplementary Figures

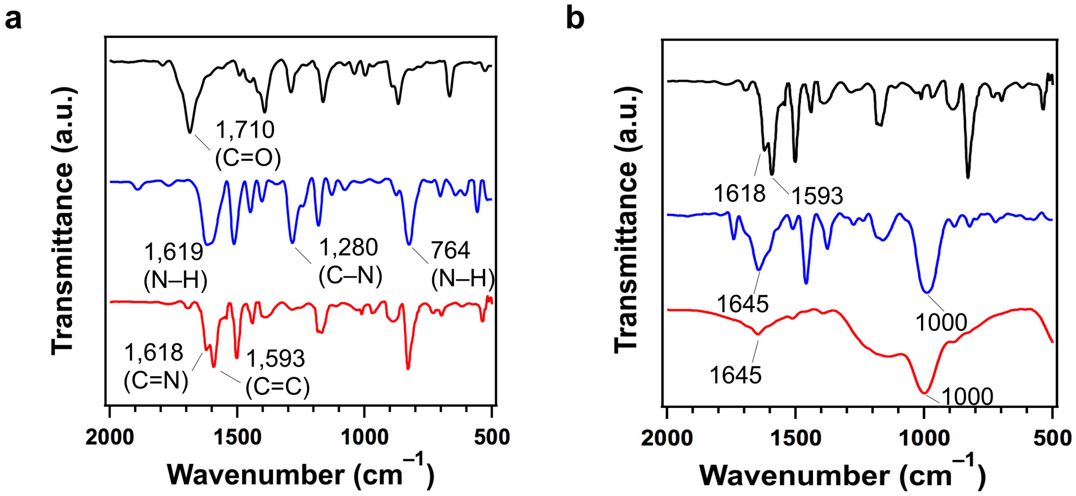


**Supplementary Figure 1. Fourier transform infrared spectra. a**, FT IR spectra of TPB (blue curve), DMeTP (black curve) and TPB-DMeTP-COF (red curve). **b**, FT IR spectra of TPB-DMeTP-COF (black curve), TPB-DMeTP-COF with $H_3PO_4$ at 2:1 molar ratio to the C=N units (blue curve) and $H_3PO_4$@TPB-DMeTP-COF (red curve).

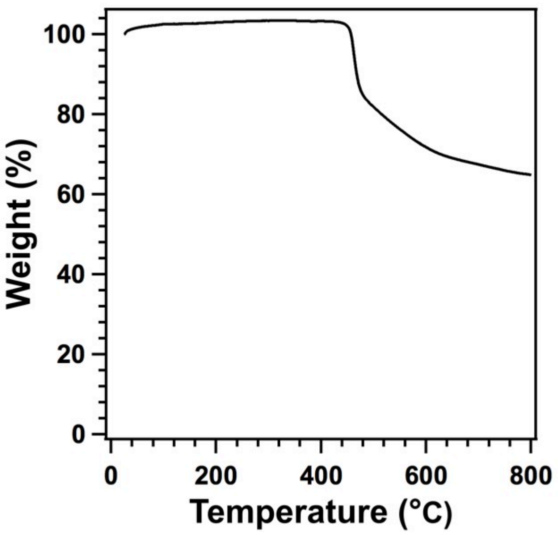


**Supplementary Figure 2. Thermogravimetric analysis.** Thermal gravimetry profile of TPB-DMeTP-COF under nitrogen.

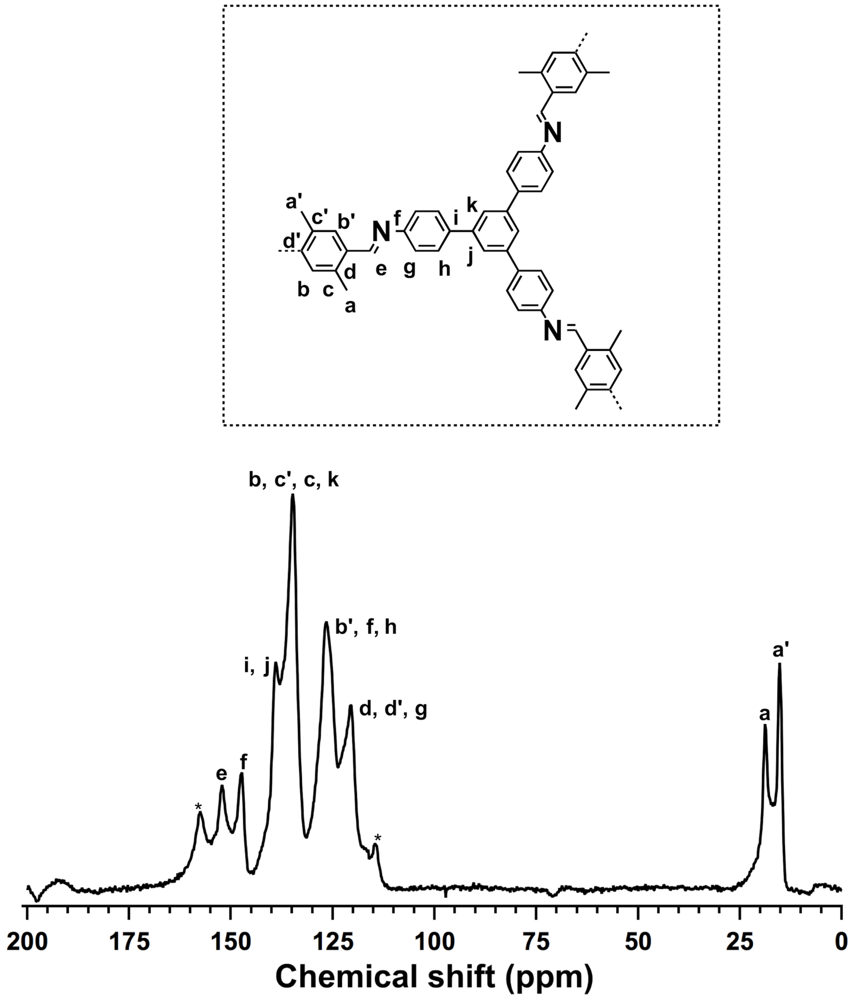


**Supplementary Figure 3. Solid-state $^{13}C$ CP/MAS spectrum.** Solid-state $^{13}C$ CP/MAS NMR spectrum of TPB-DMeTP-COF and peak assignment. The peak (e) at 157.5 ppm is assigned to the carbon of the imine C=N linkage.

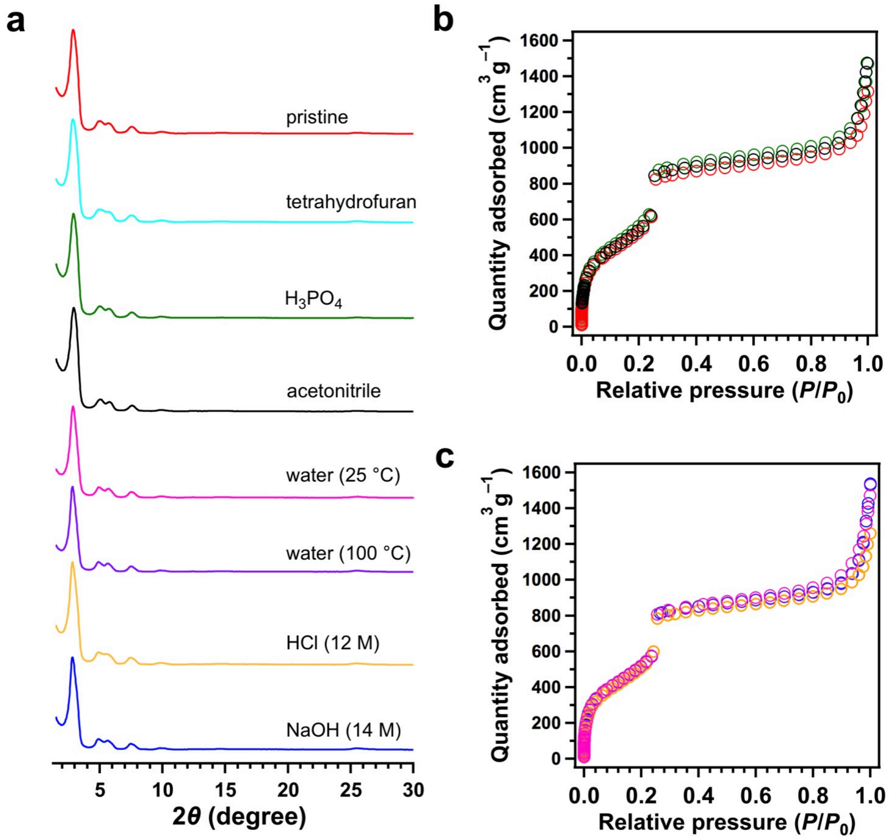


**Supplementary Figure 4. Stability. a**, PXRD patterns of as-synthesized TPB-DMeTP-COF (red curve) and TPB-DMeTP-COF after treatments in tetrahydrofuran (sky blue curve), $H_3PO_4$ (green curve), acetonitrile ($CH_3CN$, black curve), water (pink curve), boiling water (purple curve) or aqueous HCl (12 M; orange curve) and NaOH (14 M; blue curve) solutions for 7 days. **b**, Nitrogen adsorption isotherms of TPB-DMeTP-COF samples measured at 77 K after treatment in tetrahydrofuran (sky blue curve), $H_3PO_4$ (green curve), or acetonitrile (black curve) for 7 days; the red circles are for as-synthesized TPB-DMeTP-COF. **c**, Nitrogen adsorption isotherms of TPB-DMeTP-COF samples measured at 77 K upon treatment in water (pink curve), boiling water (purple curve) or aqueous HCl (12 M; orange curve) and NaOH (14 M; blue curve) solutions for 7 days.

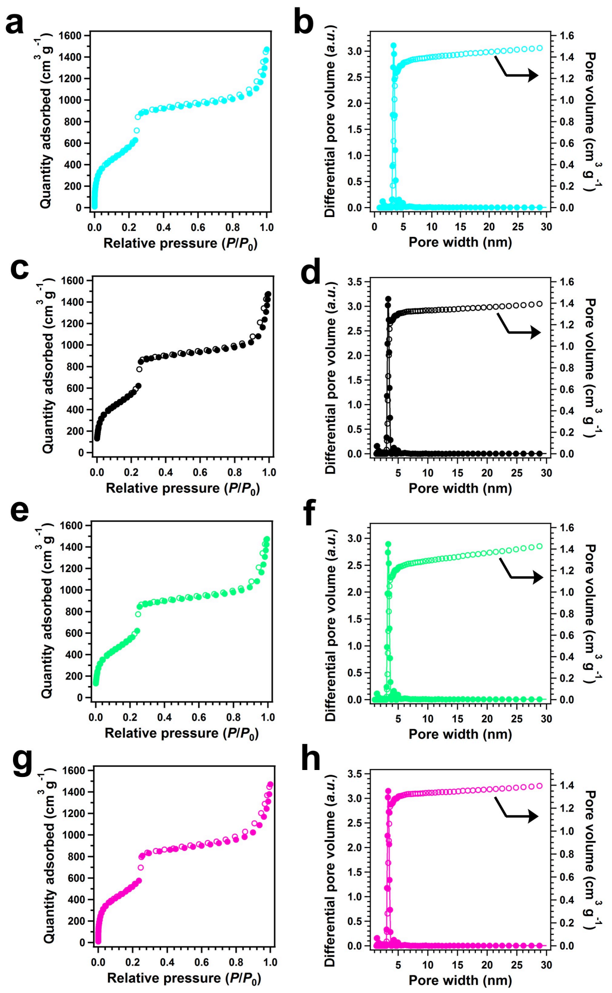


**Supplementary Figure 5. Stability. a-h**, Nitrogen sorption isotherm curves (**a**, **c**, **e** and **g**), pore size distribution and pore volume profiles (**b**, **d**, **f** and **h**) of TPB-DMeTP-COF measured at 77 K after 7-day treatment in tetrahydrofuran (**a** and **b**), $CH_3CN$ (**c** and **d**), $H_3PO_4$ (**e** and **f**) or water (**g** and **h**). Open circles are adsorption data points and filled circles are desorption data points in (**a**), (**c**), (**e**) and (**g**).

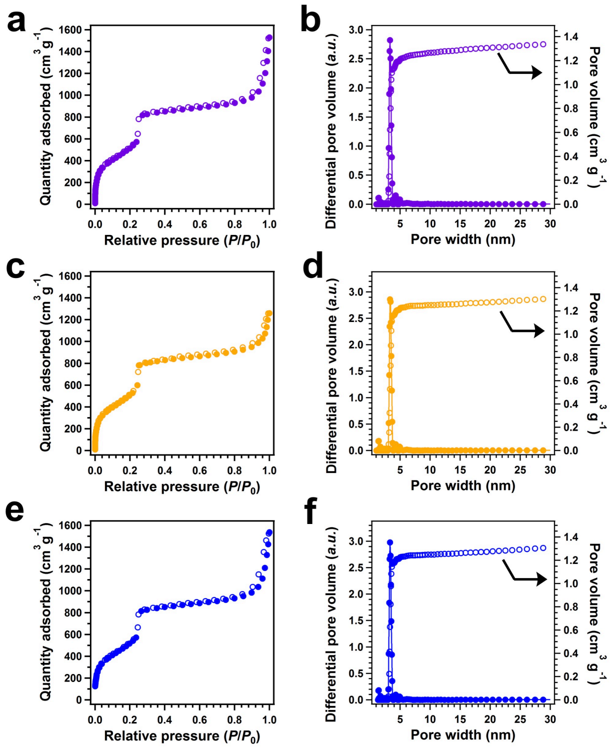


**Supplementary Figure 6. Stability. a-f**, Nitrogen sorption isotherm curves (**a**, **c** and **e**), pore size distribution and pore volume profiles (**b**, **d** and **f**) of TPB-DMeTP-COF measured at 77 K after 7-day treatment in boiling water (**a** and **b**) or aqueous HCl (12 M; **c** and **d**) and NaOH (14 M; **e** and **f**) solutions. Open circles are adsorption data points and filled circles are desorption data points in (**a**), (**c**) and (**e**).

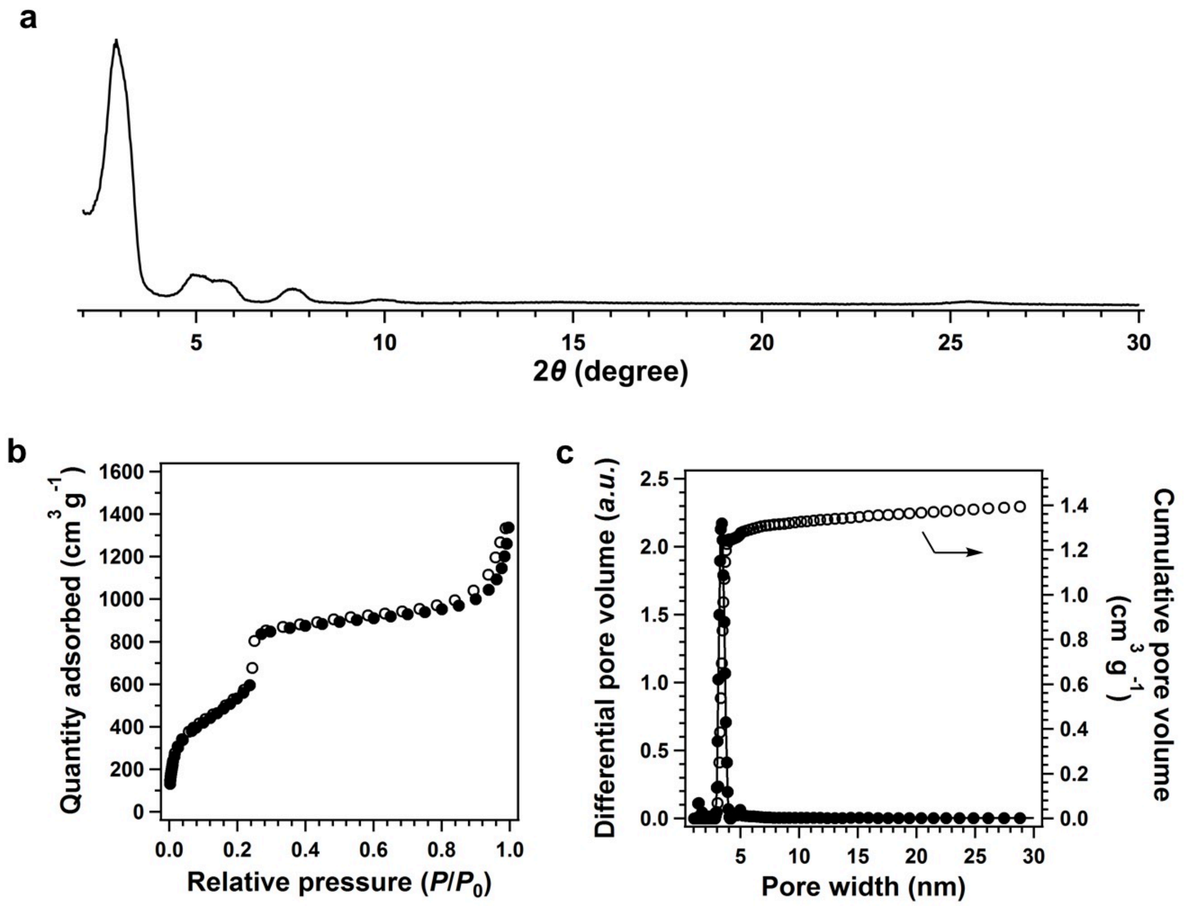


**Supplementary Figure 7. Fenton test. a**, PXRD pattern of TPB-DMeTP-COF after Fenton test. **b**, Nitrogen sorption isotherm curves of TPB-DMeTP-COF after Fenton test. Open circles are adsorption data points and filled circles are desorption data points. **c**, Pore size distribution and pore volume profiles of TPB-DMeTP-COF after Fenton test.

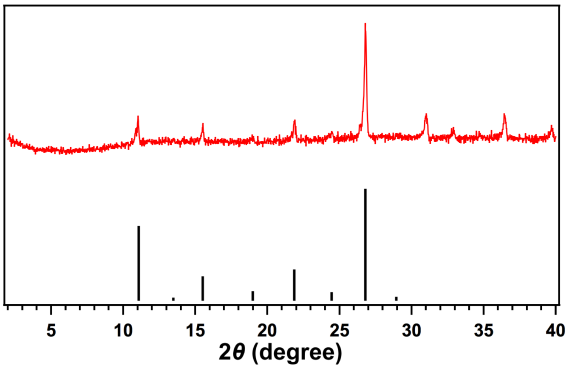


**Supplementary Figure 8. PXRD curves of $(NH_4)_3PO_4·12MoO_3$.** PXRD patterns of $(NH_4)_3PO_4·12MoO_3$ (red curve) and standard PDF card of $(NH_4)_3PO_4·12MoO_3$ (black curve).

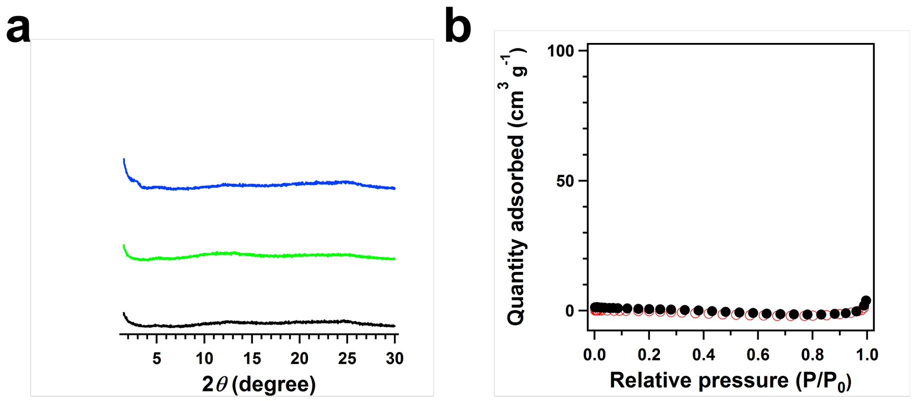


**Supplementary Figure 9. Crystallinity and Porosity. a**, PXRD pattern of $H_3PO_4$@TPB-DMeTP-COF (black curve), 75%$H_3PO_4$@TPB-DMeTP-COF (green curve) and 50%$H_3PO_4$@TPB-DMeTP-COF (blue curve) and. **b**, Nitrogen sorption isotherm curves of $H_3PO_4$@TPB-DMeTP-COF. Open circles are adsorption data points and filled circles are desorption data points.

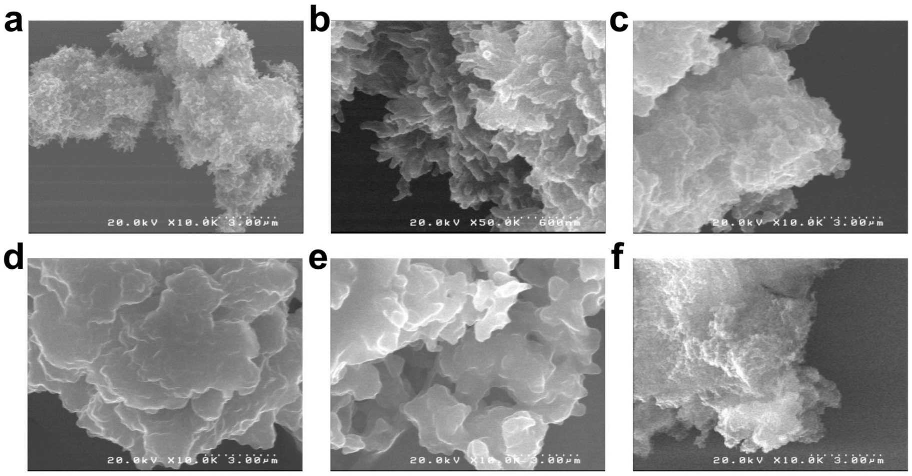


**Supplementary Figure 10. FE SEM images. a**, **b**, FE SEM images of TPB-DMeTP-COF at different magnifications. **c**, FE SEM image of 50%$H_3PO_4$@TPB-DMeTP-COF. **d**, FE SEM image of 75%$H_3PO_4$@TPB-DMeTP-COF. **e**, FE SEM image of $H_3PO_4$@TPB-DMeTP-COF. **f**, FE SEM image of TPB-DMeTP-COF after removal of $H_3PO_4$ from $H_3PO_4$@TPB-DMeTP-COF.

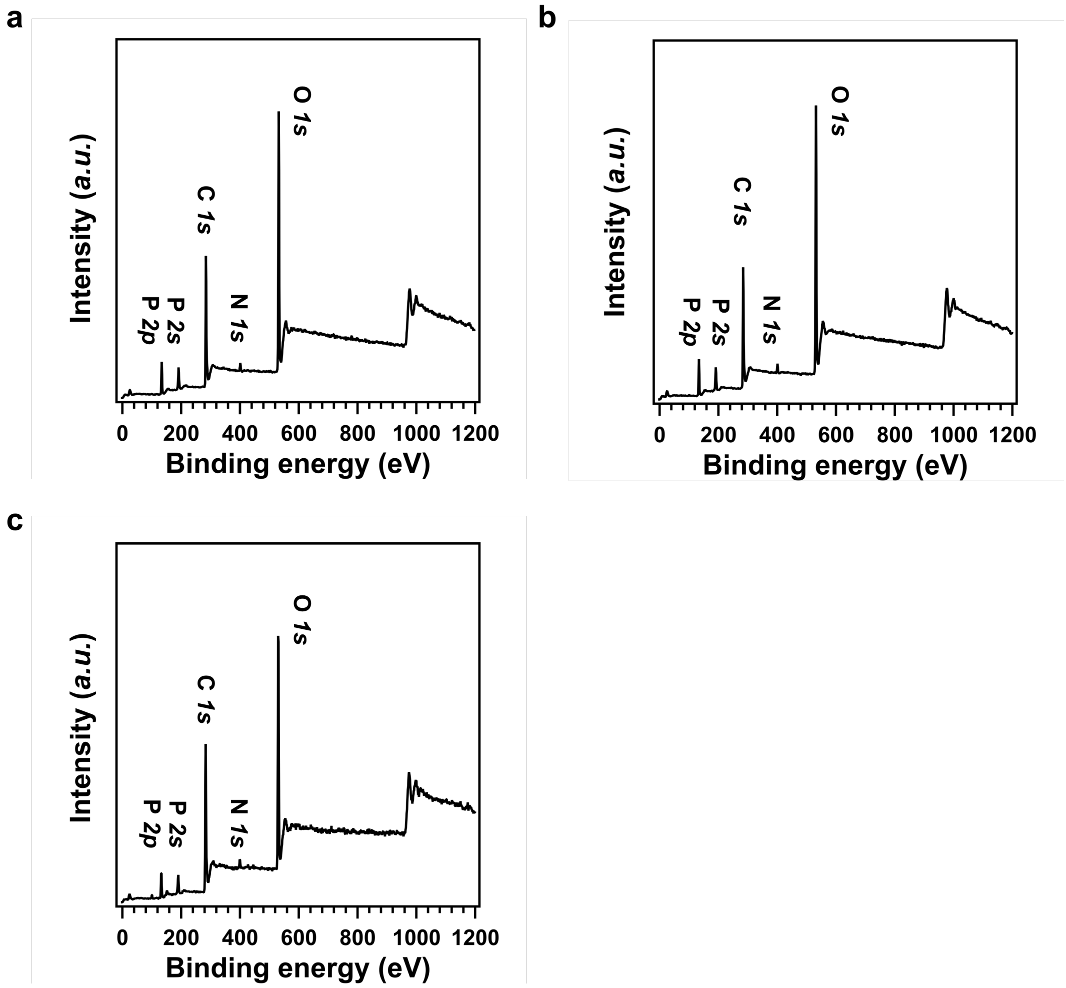


**Supplementary Figure 11. XPS analysis. a**, XPS profile of $H_3PO_4$@TPB-DMeTP-COF. **b**, XPS profile of 75%$H_3PO_4$@TPB-DMeTP-COF. **c**, XPS profile of 50%$H_3PO_4$@TPB-DMeTP-COF.

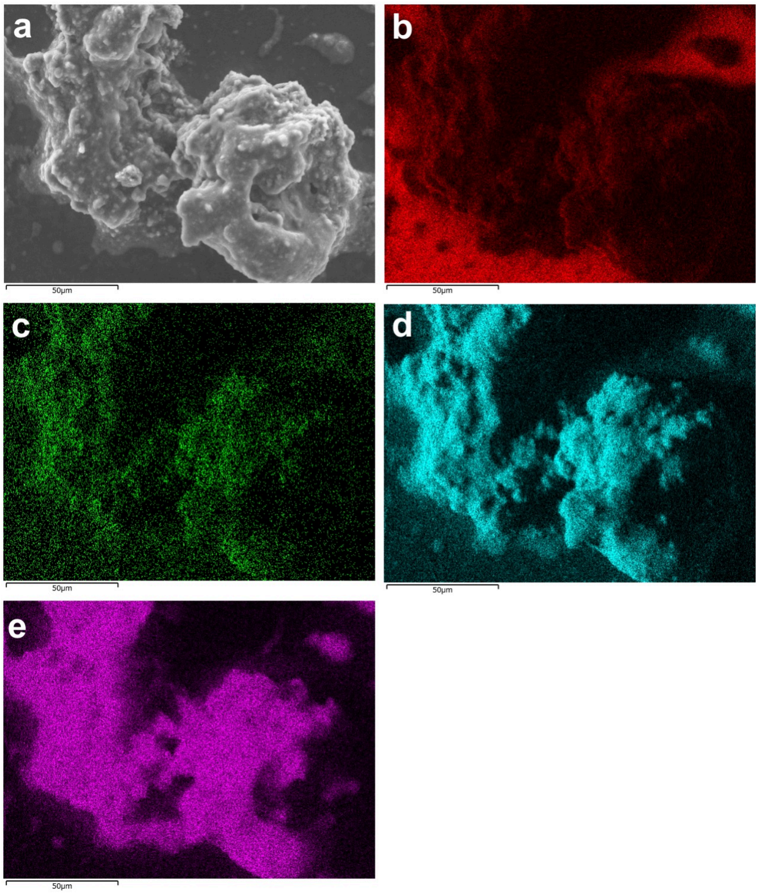


**Supplementary Figure 12. Energy dispersive X-ray spectroscopy. a,** FE SEM image of $H_3PO_4$@TPB-DMeTP-COF. (**b-e**) Elemental distributions of (**b**) carbon, (**c**) nitrogen, (**d**) oxygen, and (**e**) phosphorus.

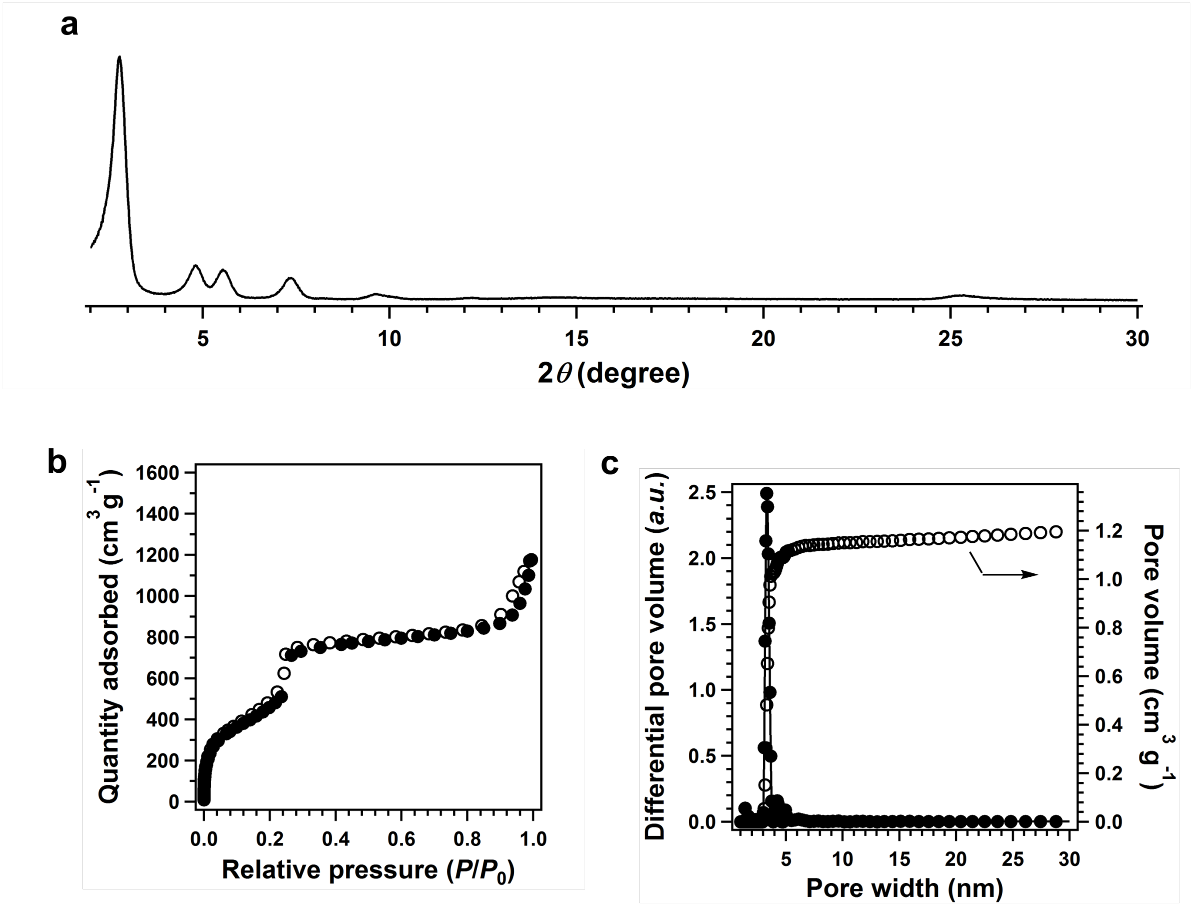


**Supplementary Figure 13. Crystallinity of porosity of TPB-DMeTP-COF after impedance measurements.** **a**, PXRD pattern of TPB-DMeTP-COF after impedance measurements. **b**, Nitrogen sorption isotherm curves of TPB-DMeTP-COF after impedance measurements. Open circles are adsorption data points and filled circles are desorption data points. **c**, Pore size distribution and pore volume profiles of TPB-DMeTP-COF after impedance measurements.

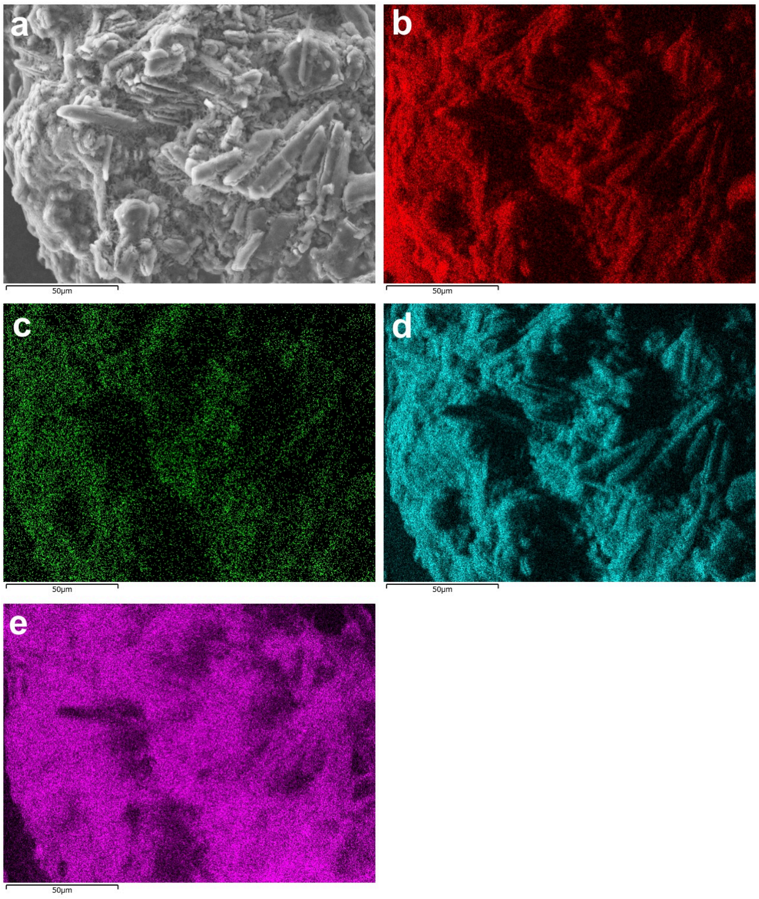


**Supplementary Figure 14. Energy dispersive X-ray spectroscopy. a,** FE SEM image of 75%$H_3PO_4$@TPB-DMeTP-COF. (**b-e**) Elemental distributions of (**b**) carbon, (**c**) nitrogen, (**d**) oxygen and (**e**) phosphorus.

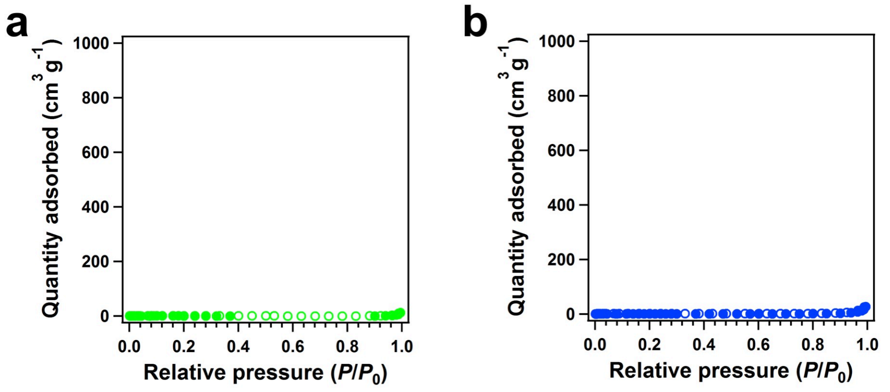


**Supplementary Figure 15. Porosity. a**, Nitrogen sorption isotherm curves of 75%$H_3PO_4$@TPB-DMeTP-COF. **b**, Nitrogen sorption isotherm curves of 50%$H_3PO_4$@TPB-DMeTP-COF. Open circles are adsorption data points and filled circles are desorption data points.

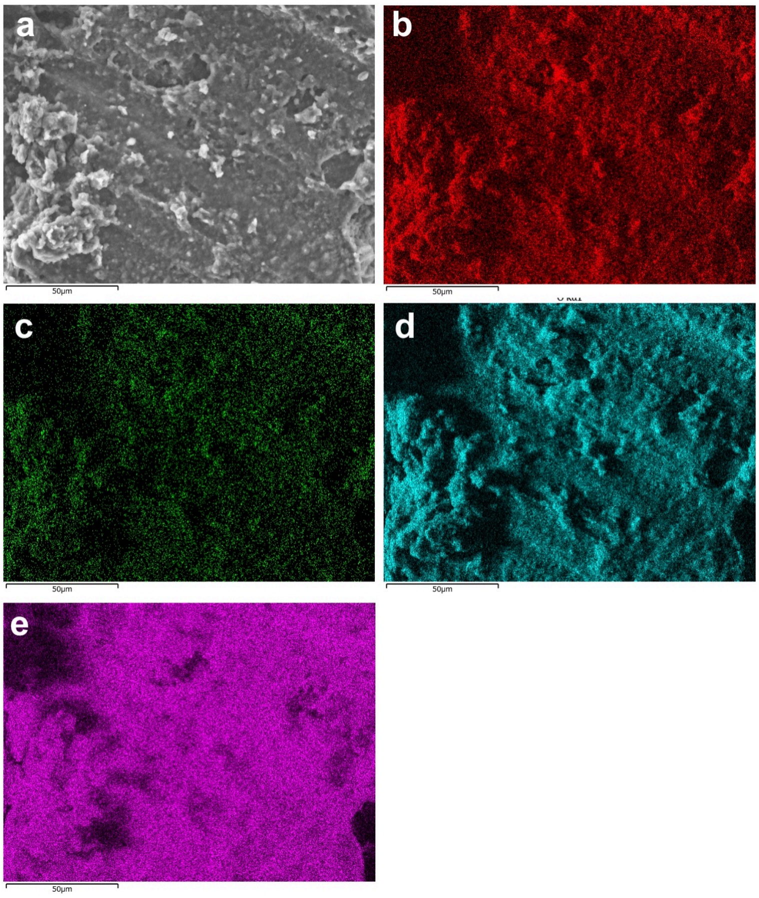


**Supplementary Figure 16. Energy dispersive X-ray spectroscopy. a,** FE SEM image of 50%$H_3PO_4$@TPB-DMeTP-COF. (**b-e**) Elemental distributions of (**b**) carbon, (**c**) nitrogen, (**d**) oxygen and (**e**) phosphorus.

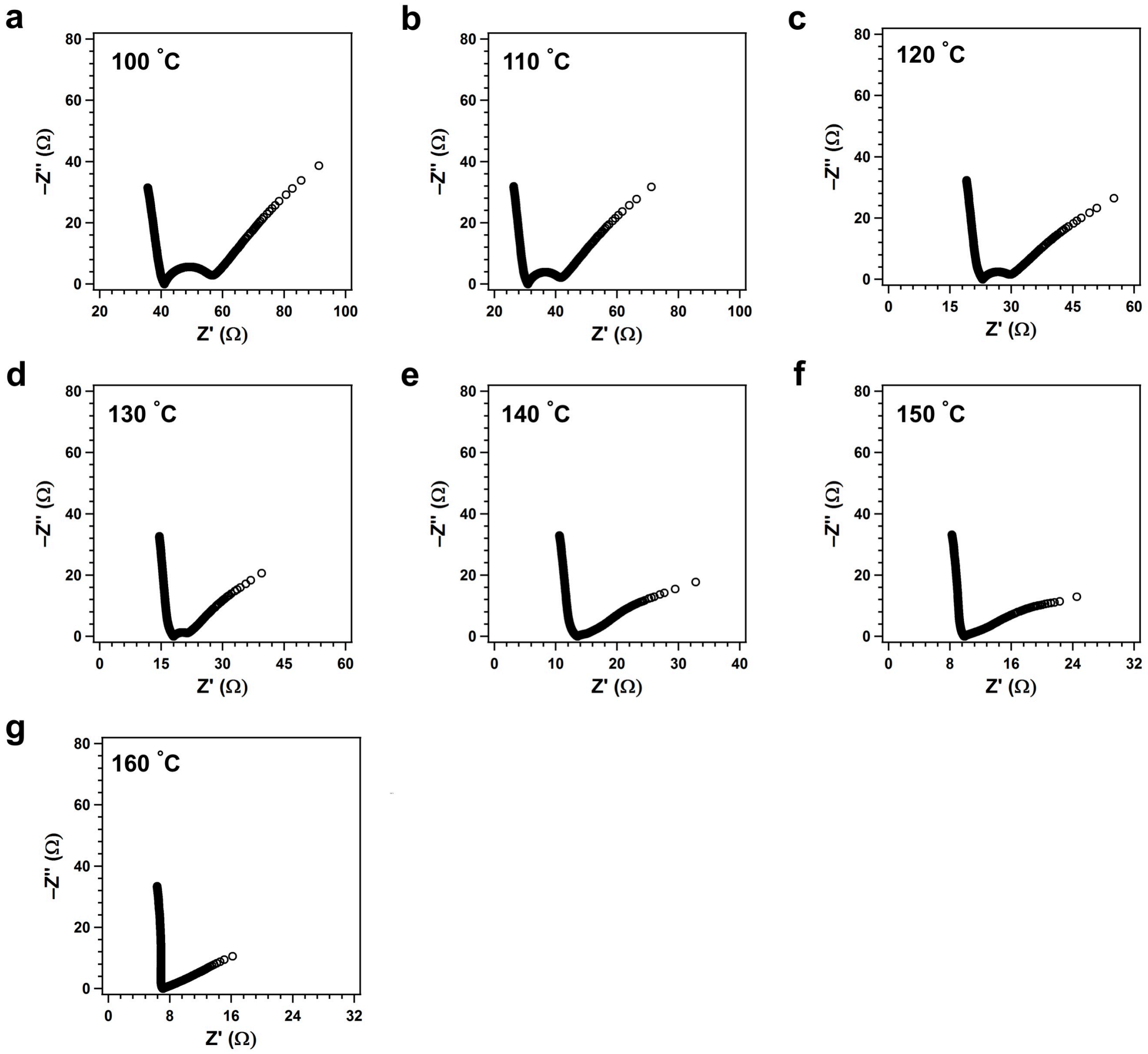


**Supplementary Figure 17. Impedance spectroscopy. a-g**, Nyquist plots of 75%$H_3PO_4$@TPB-DMeTP-COF measured at different temperatures.

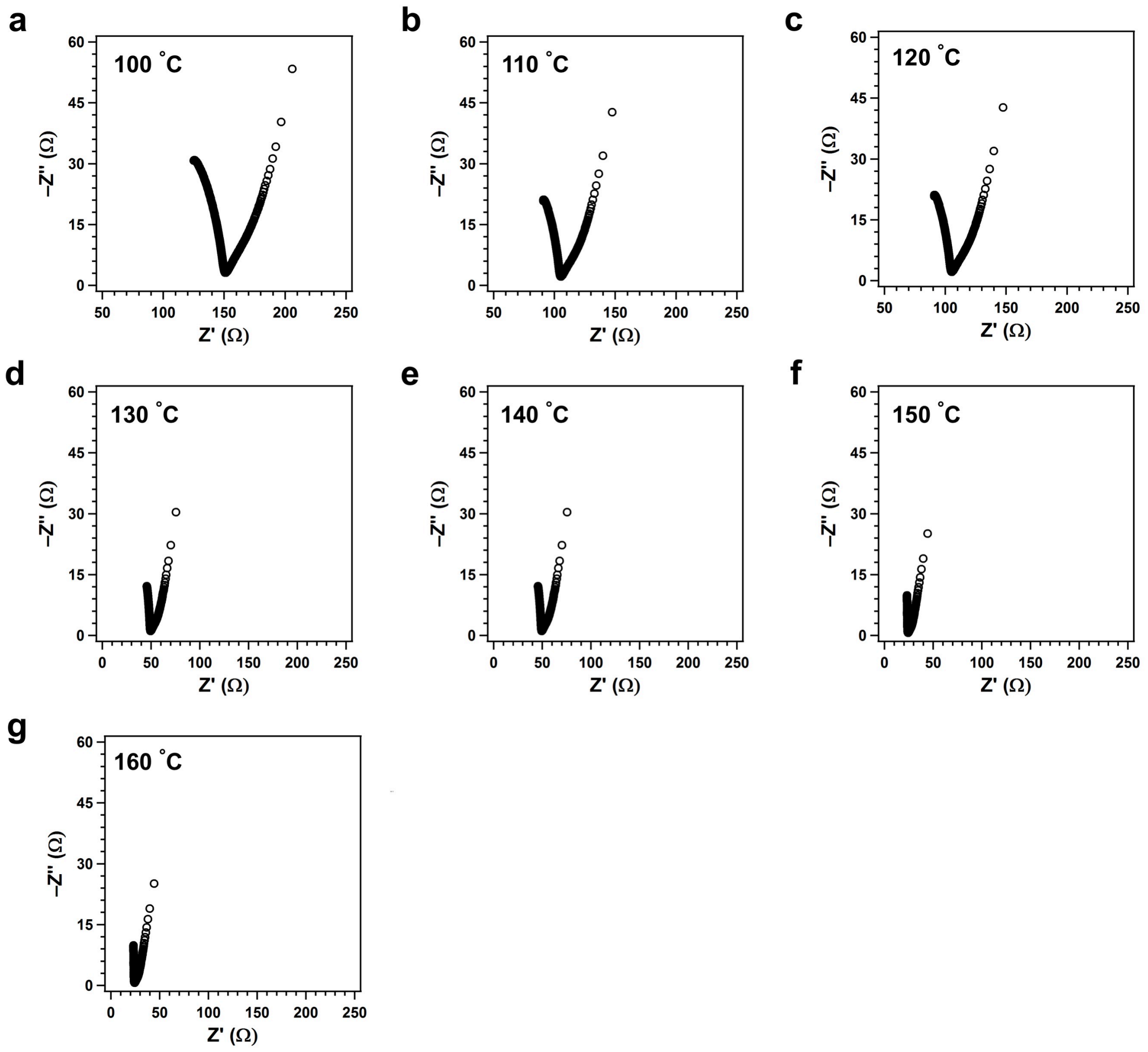


**Supplementary Figure 18. Impedance spectroscopy. a-g**, Nyquist plots of 50%$H_3PO_4$@TPB-DMeTP-COF measured at different temperatures.
